# Artificial light at night (ALAN) causes shifts in soil communities and functions

**DOI:** 10.1098/rstb.2022.0366

**Published:** 2023-12-18

**Authors:** Simone Cesarz, Nico Eisenhauer, Solveig Franziska Bucher, Marcel Ciobanu, Jes Hines

**Affiliations:** ^1^ German Centre for Integrative Biodiversity Research (iDiv) Halle-Jena-Leipzig, 04103 Leipzig, Germany; ^2^ Institute of Biology, Leipzig University, Leipzig 04109, Germany; ^3^ Institute of Ecology and Evolution with Herbarium Haussknecht and Botanical Garden, Department of Plant Biodiversity, Friedrich Schiller University Jena, Jena 07743, Germany; ^4^ Institute of Biological Research, Branch of the National Institute of Research and Development for Biological Sciences, 48 Republicii Street, 400015 Cluj-Napoca, Romania

**Keywords:** light pollution, biodiversity–ecosystem functioning, bioindicator, belowground

## Abstract

Artificial light at night (ALAN) is increasing worldwide, but its effects on the soil system have not yet been investigated. We tested the influence of experimental manipulation of ALAN on two taxa of soil communities (microorganisms and soil nematodes) and three aspects of soil functioning (soil basal respiration, soil microbial biomass and carbon use efficiency) over four and a half months in a highly controlled Ecotron facility. We show that during peak plant biomass, increasing ALAN reduced plant biomass and was also associated with decreased soil water content. This further reduced soil respiration under high ALAN at peak plant biomass, but microbial communities maintained stable biomass across different levels of ALAN and times, demonstrating higher microbial carbon use efficiency under high ALAN. While ALAN did not affect microbial community structure, the abundance of plant-feeding nematodes increased and there was homogenization of nematode communities under higher levels of ALAN, indicating that soil communities may be more vulnerable to additional disturbances at high ALAN. In summary, the effects of ALAN reach into the soil system by altering soil communities and ecosystem functions, and these effects are mediated by changes in plant productivity and soil water content at peak plant biomass.

This article is part of the theme issue ‘Light pollution in complex ecological systems’.

## 1. Introduction

Natural light, i.e. moon and sunlight, has been extremely consistent, with periodic recurring cycles over long periods of geological time. It is, therefore, a reliable environmental cue to which many biological systems are calibrated [1]. However, with the increasing amount of artificial light at night (ALAN), formerly consistent natural light cues are being disrupted, causing changes at multiple levels of organization [2]. Most previous studies examining the influence of ALAN have focused on the responses of aboveground animals, such as changes in abundance, life-history traits and physiology. So far, evidence showing ecosystem functions and plant-related responses to ALAN is still scarce [2]. Thereby, a comprehensive view of multiple types of responses and organizational levels is important in order to be able to evaluate the threats caused by ALAN [3,4].

Understandably, most ALAN studies focus on the responses of organisms and functions that are directly exposed to artificial light sources. Accordingly, responses of soil organisms have been excluded, despite their high biodiversity and importance for ecosystem functions such as nutrient cycling, carbon sequestration and decomposition [5–7]. Most studies on ALAN involving microorganisms—the main driver of the mentioned functions—are limited to sediments in freshwater systems [8–11]. Since the soil system harbours enormous biodiversity, is responsible for main ecosystem functions and services and is irretrievably connected to the aboveground system [5,12,13], knowing the consequences of ALAN for the belowground system is of high interest.

In soil, the effects of ALAN could operate indirectly through the plant community via root exudation [14,15], leaf litter [16] and microclimatic conditions [17,18]. Changes in belowground resource input rates can be due to plant shoot physiological responses, e.g. increased photosynthesis leading to higher plant biomass or oxidative stress leading to reduced photosynthetic rates and carbon and nitrogen starvation [19]. Also, changes in soil microclimatic conditions can stem from reduced stomatal functioning caused by ALAN, which limits water-use efficiency [20]. In turn, the quantity and quality of resource inputs, but also soil moisture and temperature, are the main drivers of the abundance, activity, diversity and functioning of soil organisms [21–23]. Due to this strong connection between plants and soil organisms, ALAN may significantly influence the soil system and the functions it provides, but this has not yet been studied.

Two key groups of soil biota can provide powerful information on the functioning of the soil system and may indicate soil sensitivity to ALAN. First, soil microorganisms are the main drivers of critical ecosystem functions, such as soil respiration. Changes in soil microbial biomass and respiration are often associated with warmer microclimates and increased soil moisture or resource availability [24]. Second, free-living soil nematodes are regarded as indicators of soil health and quality due to their high trophic and functional diversity [25]. Soil nematodes can be divided into bacterial, fungal, and plant feeders, as well as into predators and omnivores (trophic groups). In addition, each trophic group can be further divided based on life-stage history traits, which can be roughly described as a gradient of r- and K-strategists (r-strategists are species with life history characteristics making them well suited for exploiting transient environments; K-strategists are species with life history characteristics making them well suited for population stability and stable environments) [26–28]. A high number of r-strategists would indicate stressed conditions that favour individuals with rapid growth and short generation time. By contrast, high proportions of K-strategists (mainly predators and omnivores) indicate increased soil food web complexity and stable food resources that support slower growth. If ALAN imposes stress leading to reduced fitness of plants [29,30], then soil nematode community analysis would reveal the consequences for soil food webs [26–28].

Here we present one of the first experiments testing the influence of ALAN on soil community composition and ecosystem functioning. By simulating different levels of ALAN under controlled Ecotron conditions, we investigated the response of the abundance, activity and composition of the soil microbial community, as well as the abundance, composition, diversity and structural components of the nematode community. We hypothesize that (1) ALAN affects soil community composition and functioning and (2) that these effects are mediated by plant biomass and plant diversity. We further suggest that (3) potential negative impacts on plants will be reflected in the structure of soil nematode communities, such as increased proportions of r-strategists and decreased composition of K-strategists, indicating a reduction in the food web structure.

## 2. Material and methods

### (a) Experimental units

The effect of ALAN was tested in 12 experimental chambers (EcoUnits) of the iDiv Ecotron. The lower parts have dimensions 1.24 m × 1.24 m, with a depth of 0.80 m, and can hold 1.23 m³ of soil [31]. The upper parts of the EcoUnits have dimensions 1.46 m × 1.46 m, with a height of 1.50 m. An irrigation system in the aboveground part allowed controlled irrigation of the whole area. Daylight was applied with a diffuser that held four LED lamps. During the day, the light was brought to 350 µmol m^2^ s^−1^ (corresponding to 83.58 lux using a conversion factor of 0.2388). Drainage of water took place via four suction systems installed at the bottom of the Ecounit, leading to a negative pressure simulating natural water drainage.

### (b) Light regime at night

At night, moonlight within each EcoUnit was simulated by a single sunlike LED (SunLike3030 by Seoul Semiconductor Co. Ltd., Korea). Moonlight intensities were modelled for the real-time and location of the experiment. The illuminance of the moonlight, i.e. the background light regime that followed near-natural cycles from new to full moon cycles during the night, was adjusted every minute using a Python script and could be adjusted to 57 illuminance levels ranging from 0 lux (off) to the maximum modelled moonlight brightness of around 0.274 lux. Additionally, to simulate ALAN, we used LED lights with a typical blue light peak (type 2835 by HuiYuan Opto-Electronic Co. Ltd., China). We generated ten different levels of artificial light on a log_10_ scale, starting at 0.0014 lux, corresponding to the illuminance of starlight, and 0.0087, 0.028, 0.081, 0.10, 0.30, 0.94, 3.03, 9.88, up to 30.31 lux (in the absence of moonlight), comparable to typical street lighting. The lowest and highest light levels were replicated twice. The lower half of the ALAN illuminance levels were below the maximum brightness of the full moon during the experimental phase. The treatment lights were scattered using diffusion foil and turned on and off gradually at sunrise and sunset, respectively, to avoid startling aboveground insects (see [32]). All EcoUnits were covered with black theatre curtains to prevent external light sources from influencing the light regime. Illuminance levels of all lights at night were calibrated using a sky brightness measurement approach with a fisheye-lens camera [33].

### (c) Soil and plant community

The EcoUnits were filled with loamy sand soil (sand: 84%, silt: 11%, clay: 5%) from the vicinity of the iDiv Ecotron, with a soil pH of 7.4, 0.83% of total carbon and 0.06% of total nitrogen. Before filling the soil into the EcoUnits, it was well mixed and homogenized but not sterilized. After a settling period of about five months, the soil density was 0.20 g cm^−^³. The plant community consisted of 16 grassland species and was sown and exposed to the experimental light regime as described in [34]. Some plant species of the seed bank were introduced as well. A total of five cuts of plant biomass were made at different intervals (electronic supplementary material, figure S1) to simulate intensive grassland management in the region [35]. Only the last two cuts were analysed for plant biomass, plant species composition and diversity, as well as plant functional traits [34].

### (d) Experimental duration and setup

From packing the EcoUnits with soil until the first soil sampling, 195 days (approx. 6.5 months) passed (electronic supplementary material, figure S1). ALAN treatments and lunar cycles were applied from day 165 (after approx. 5.5 months) onwards and lasted for more than 4.5 months, corresponding to the duration of the experimental study. Each of the lunar cycles was 28 days long. At the beginning of the second, fourth and sixth lunar cycles, soil samples were taken; with almost two months between each soil sampling event, this resultedg in three distinct phases (phases 1, 2, and 3). Plant biomass was removed in irregular intervals after 125, 147, 203, 263 and 305 days. On the three dates of soil sampling, plant biomass harvesting occurred shortly thereafter, i.e. 8, 12 days, and immediately (= 0 days) after the soil sampling events, respectively.

### (e) Belowground measurements

At the end of each lunar cycle, four soil samples consisting of 2 cm diameter cores were taken and pooled from the top at 10 cm depth in each quarter of the EcoUnits. An O_2_-micro-compensation apparatus was used to measure soil basal respiration and active soil microbial biomass [36]. Soil basal respiration is the respiration of the soil without any addition of water and nutrients and represents mainly the respiration of soil microorganisms at the time of sampling. It is only a fraction of the whole living soil microbial biomass, as not all microorganisms find conditions that allow activity. By contrast, the total soil microbial biomass consists of all microorganisms that are alive, active and inactive. Inactive stages can be activated and then measured by the provisioning of water and glucose as substrates. The soil microbial biomass reflects conditions over longer periods of time, whereas basal soil respiration reflects short-term conditions. Measurements were done with around 7 g of fresh soil sieved at 2 mm to remove stones, large organic materials like roots and litter, as well as larger organisms. In the first step, soil basal respiration was measured for 24 h. The mean of the last 10 h, when respiration was stable, was used to calculate soil basal respiration. Afterwards, each sample received 24 mg of glucose and water needed to reach a moisture level of 20% of the soil's fresh weight. The three lowest consecutive respiration readings during the first 10 h were used to calculate soil microbial biomass, as it is assumed that no growth has yet taken place within the first 10 h. This value was multiplied by 38, a calibration factor that removed differences between the main methods to measure microbial biomass and allowed for comparison [37]. At the end of the measurements, the soil water content was estimated via drying.

Soil microbial community composition was analysed using phospholipid fatty acid (PLFA) analysis [38,39]. It is based on the concept that specific PLFAs, the main components of cell membranes, can only be synthesized by one group, making them a reliable biomarker, although different approaches to PLFA assignment exist [40,41]. We followed the protocol by Frostegård *et al*. [38] to extract fatty acid methyl esters (FAME) and measured them on a GC-FID (Clarus 680, PerkinElmer, Waltham, USA; carrier gas helium; flame ionization detector) coupled to a SP-2560 capillary column (100 m × 0.25 mm i.d., 0.2 µm film thickness). Based on Ruess and Chamberlain [40], we used the following fatty marker acids (from the PLFA fraction if not stated differently) in our analysis: arbuscular mycorrhizal fungi (AMF) marker: 20:1*ω*9, and 16:1*ω*5 from the NLFA fraction; bacterial marker: G-positive: a15:0, i15:0, i16:0, i17:0; gram-negative: cy17:0, cy19:0; fungal marker: 18:1*ω*9, 18:2*ω*6,9, 18:3*ω*3,6,9, 18:3*ω*6,9,12; unspecified microbial marker: 16:1*ω*5; and unspecific bacteria marker: 16:1*ω*7.

Nematodes were extracted from 25 g of fresh soil, which was sieved at 2 mm to increase extraction efficiency and lasted for 72 h [42], and fixed in 4% formalin. Increasing the amount of soil was not possible based on repeated measurements and further samples. However, when the Baermann funnel method was tested using 25 g of soil, it resulted in the highest extraction efficiency [42]. All nematodes in one sample were counted at 50× magnification, but if the number of nematodes in one sample exceeded 1000, only 100 individuals (or 10%) were identified using an inverse microscope with DIC (Leica DMI 4000B) at 1000× magnification. Free-living soil nematodes were used as soil bioindicators to assess soil biodiversity (measured as genus richness) and community composition of bacterial feeders, fungal feeders, plant feeders, omnivores and predators [43]. In addition, nematodes can be used to calculate different functional indices. The channel index indicates the dominant decomposition pathway based on the abundance of opportunistic fungal-feeding nematodes and bacterial-feeding nematodes in the community. Low Chanel Index values indicate a bacterial-dominated decomposition pathway, whereas high values refer to a more fungal-dominated system [26]. Information about food web complexity can be derived from the structure index. High values indicate a higher degree of K-strategists and a more stable food web. The calculation incorporates weighted abundances of cp-3 (r-strategists' tendencies) to cp-5 nematodes (pure K-strategists) of all major nematode trophic groups except plant feeders (i.e. bacterial feeders, fungal-feeders, predators and omnivores [26–28]). Higher values of the structure index indicate a higher food web complexity (i.e. higher abundances and numbers of taxa following a K-strategy [26,27]).

### (f) Statistical analysis

Although the ALAN treatment was designed as a gradient, the distribution of residuals did not meet assumptions for a linear analysis so the light pollution gradient was divided into low (*n* = 6) and high (*n* = 6) light pollution. Subsequently, we used mixed-effects linear models with ALAN (2 levels: low and high), PHASE (3 levels: phases 1, 2 and 3) and the interaction as fixed factors, and EcoUnit as a random factor to account for non-independence of repeated samples within EcoUnits using the lme4 package [44]. Data were checked for linearity, normal distribution of residuals and homogeneity of variance and log_10_-transformed. In some cases, single outliers were removed. The Kenward-Roger method corrected for multiple comparisons was used to perform a *post-hoc* test using the emmeans function from the emmeans package [45].

We analysed the microbial and nematode community composition using the biomasses of single PLFA and NLFA markers (as nanogram fatty acid marker per gram dry soil) and the relative abundance of identified nematode genera, respectively. Nonmetric multidimensional scaling (NMDS) was employed to visualize the community composition using the metaMDS function from the vegan package [46]. Bray-Curtis dissimilarity index was used to calculate a two-dimensional distance matrix with a maximum of 100 random starts to obtain a stable solution. We chose Bray-Curtis as it can handle abundance data and zeros. However, at the same time, using Bray-Curtis can have disadvantages, e.g. being sensitive to rare species and not accounting for species identity. As no index can encompass all ecological aspects [47] and different indices may be required to address various ecological questions, we believe that Bray-Curtis dissimilarities provide a good convergence to describe changes in a community. Significance of the community composition vectors was assessed using the envfit function from the vegan package with 999 permutations, considering only those vectors with a significance level of *α* = 0.05. To examine the differences between the factors ALAN, PHASE, and their interaction, we employed the adonis2 function from the vegan package [48], which conducts a permutation test with pseudo F-ratios on distance matrices. To determine specific group differences (e.g. high ALAN in phase 1 versus low ALAN in phase 3), a PERMANOVA analysis using the adonis2 function was performed on a reduced dataset focused on the specific contrast of interest. All statistical analyses were conducted using R, version 4.1.2 [49].

## 3. Results

We found that soil microbial and nematode communities are different under different levels of ALAN. However, the specific responses to ALAN, including their presence, magnitude and direction, depended on the particular response variable being considered and the experimental phase. The effect of ALAN also had an impact on plant biomass in phase 2, but our response variables were not affected by plant biomass in this phase. By contrast, in phase 3, when plant biomass was not significantly affected by ALAN, three out of 14 response variables were affected by high ALAN.

**Figure 1.**
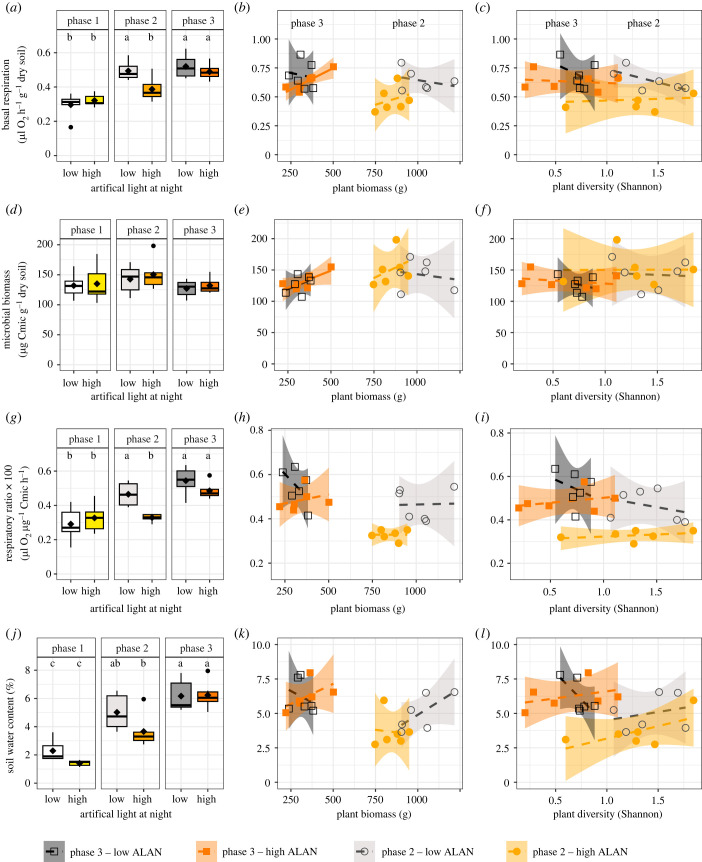
The effect of artificial light at night (low and high) and time (phase 1 to 3, representing one lunar cycle each), plant biomass and plant diversity as indicated by the Shannon diversity index on (*a–c*) soil basal respiration, (*d–f*) soil microbial biomass, (*g–i*) the respiratory ratio, i.e. the ratio of basal respiration and microbial biomass indicating carbon use efficiency (low values indicate a higher carbon use efficiency) and (*j–l*) soil water content. Letters above boxplots show results of a *post hoc* test for the significant result of a linear mixed effect model. Different letters indicate significant differences at *α* = 0.05. Dashed lines in correlations indicate non-significant relationships, and the solid lines ((*b*) and (*e*) for high ALAN) have a marginal significant effect indicating an ecological trend at *α* < 0.10.

Among the three soil-microbial properties examined, two of them demonstrated a significant interaction between ALAN and the experimental phase (table 1). That is, soil basal respiration and the respiratory ratio, but not soil microbial biomass, increased significantly with time. But only in phase 2—the phase with highest plant biomass—were the different levels of ALAN significantly different from each other. At higher ALAN, basal respiration and the respiratory ratio decreased (figure 1*a,g*). A similar trend was observed for soil water content, although the *post hoc* test did not support significant difference between low and high ALAN in phase 2 (table 1, figure 1*j*). Soil microbial biomass did not exhibit significant changes over time or in response to ALAN (figure 1*d*).

**Table 1.** ANOVA table of linear mixed-effects models testing for the effect of ALAN (low and high) and three consecutive lunar cycles (PHASE: 1, 2 and 3) and its interaction on soil microbial properties and different parameters based on nematode community composition. Significant results at *α* < 0.5 are indicated with asterisks (levels see below), and italics indicate marginal significant results indicating ecological trends at *α* < 0.1.

	variable	factor	NumDF	DenDF	*F*	*P*
soil microbial properties and soil water content	basal respiration	ALAN	1	10	2.18	0.171
		PHASE	2	20	51.84	<0.001***
		AxP	2	20	5.72	0.011*
	microbial biomass	ALAN	1	10	0.35	0.569
		PHASE	2	20	2.13	0.145
		AxP	1	20	0.06	0.941
	respiratory ratio	ALAN	1	10	5.01	0.033*
		PHASE	2	20	25.04	<0.001***
		AxP	2	20	4.38	0.021*
	soil water content	ALAN	1	10	6.33	0.031*
		PHASE	2	20	112.30	<0.001
		AxP	2	20	3.46	*0.051.*
nematode trophic group parameters	total nematode abundance	ALAN	1	10	0.06	0.813
		PHASE	2	20	48.68	<0.001***
		AxP	2	20	0.30	0.743
	bacterial feeders (BF)	ALAN	1	10	0.28	0.610
		PHASE	2	20	28.04	<0.001***
		AxP	2	20	0.10	0.909
	fungal feeders (FF)	ALAN	1	10	0.84	0.381
		PHASE	2	20	13.89	<0.001***
		AxP	2	20	1.11	0.350
	plant feeders (PF)	ALAN	1	10	3.95	*0.056.*
		PHASE	2	20	36.26	<0.001***
		AxP	2	20	1.37	0.589
	omnivores + predators (OM + PR)	ALAN	1	10	0.92	0.361
		PHASE	2	20	18.86	<0.001***
		AxP	2	20	1.54	0.238
	r-strategists	ALAN	1	10	0.17	0.681
		PHASE	2	20	28.65	<0.001***
		AxP	2	20	0.10	0.906
	K-strategists	ALAN	1	10	1.03	0.334
		PHASE	2	20	47.43	<0.001***
		AxP	2	20	1.87	0.181
nematode indices	nematode diversity (Shannon index)	ALAN	1	10	0.09	0.766
		PHASE	2	20	3.75	0.041*
		AxP	2	20	2.27	0.130
	channel index (decomposition channel)	ALAN	1	10	9.52	0.004**
		PHASE	2	20	5.52	0.009**
		AxP	2	20	1.20	0.318
	structure index	ALAN	1	10	0.10	0.754
		PHASE	2	20	4.94	0.018*
		AxP	2	20	1.82	0.189

*** = *p* < 0.001; ** = *p* < 0.01; * = *p* < 0.05; **.** = *p* < 0.1.

During phase 2, plant biomass was generally higher compared to phase 3 (for more details, see [34]). Interestingly, high levels of ALAN resulted in a reduction in plant biomass compared to low levels of ALAN (figures 1*b,e,h,k*). However, these differences in plant biomass did not lead to any significant correlations with the soil microbial properties and soil water content, even though high and low levels of ALAN induced distinct patterns (electronic supplementary material, table S1). In phase 3, where plant biomass was lower compared to phase 2 and more time had passed, basal respiration increased significantly and microbial biomass marginally significantly with plant biomass, but only under high levels of ALAN. Surprisingly, plant diversity did not significantly impact the soil microbial properties or soil water content (figures 1*c,f,i,l*; electronic supplementary material, table S1).

**Figure 2.**
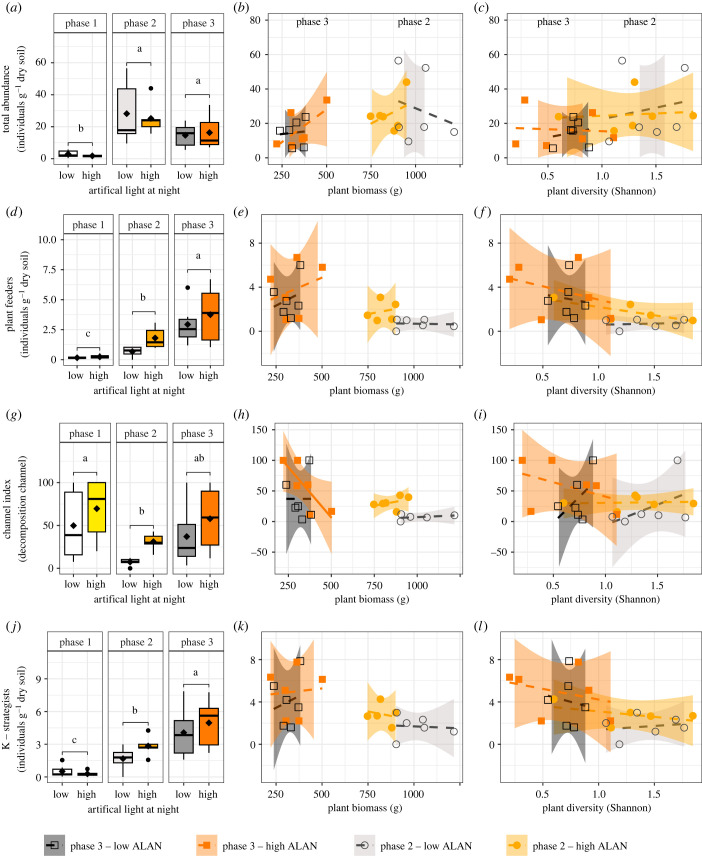
The effect of artificial light at night (low and high) and time (phase 1 to 3, representing one lunar cycle each), plant biomass, and plant diversity as indicated by the Shannon diversity index on (*a–c*) Total nematode, (*d–f*) plant-feeding nematodes (*g*–*i*) the channel index that provides information on the dominant decomposition channel (higher values indicate a higher relevance of fungal decomposition, and (*j–l*) the abundance of K-strategists. Letters above boxplots show results of a *post hoc* test for the significant result of a linear mixed effect model. Different letters indicate significant differences at *α* = 0.05. Dashed lines in correlations indicate non-significant relationships and the solid line (*h*) for high ALAN) has a marginal significant effect indicating an ecological trend at *α* < 0.10.

Generally, the nematode community was composed mainly of bacterial feeders, intermediate abundances of fungal and plant feeders and relatively few omnivores and predators (electronic supplementary material, figure S2). Among the treatments examined, the experimental phase (PHASE) exerted the strongest influence and significantly impacted all ten nematode parameters analysed, although the direction of the effect was not consistently the same (figure 2). Only two out of the ten nematode parameters (plant feeders and channel index) were affected by ALAN, while the interaction between PHASE and ALAN had no significant effect (table 1). Specifically, plant feeders showed a marginal increase under high compared to low ALAN (table 1; figure 2*d*). However, neither covariate (plant biomass, plant diversity) had a significant impact on plant-feeder densities (electronic supplementary material, table S1; figures 2*e,f*). Among the ten nematode parameters, no one showed a significant effect at *α* < 0.05. Only the channel index exhibited an ecological trend with plant biomass, decreasing marginally significantly under high ALAN in phase 3 (electronic supplementary material, table S1; figure 2*h*). This was due to a significant reduction of bacterial feeders in phase 3 compared to phase 2 (electronic supplementary material, figure S3A), whereas the densities of the fungal feeders remained more stable (electronic supplementary material, figure S3D). Once again, although differences were most pronounced in phase 2, a relationship with plant biomass was only observed in phase 3, and plant diversity had no effect.

The primary effect of the experimental phase (PHASE) on nematode abundances was the overall low densities of all groups during the initial phase (phase 1) compared to the later phases (figure 2*a*, electronic supplementary material, figure S2). The absolute densities of microbial-feeding nematodes (electronic supplementary material, figure S3A, D; i.e. bacterial and fungal feeders) increased after phase 1 to phase 2. As mentioned before, bacterial feeders dropped in phase 3 compared to phase 2, whereas fungal feeders did not differ between phases 2 and 3. Due to the dominance of bacterial feeders, r-strategists, which include only bacteria and fungivores, showed the same trend as bacterial feeders (electronic supplementary material, figure S3M). This pattern contrasts with the response of K-strategists (figure 2*j*) and plant feeders (figure 2*d*), which both exhibited significant increases with each phase. Similarly, the combination of omnivores and predators (electronic supplementary material, figure S3G) and the structure index (electronic supplementary material, figure S3P) increased with time. The nematode diversity, as measured by the Shannon diversity index, was found to be lowest in phase 2 when plant biomass was high and differed significantly from phase 1 (electronic supplementary material, figure S3J). However, there was no significant change in nematode diversity from phase 1 to phase 3, suggesting that this decrease did not lead to an overall alteration in nematode diversity over time.

**Figure 3.**
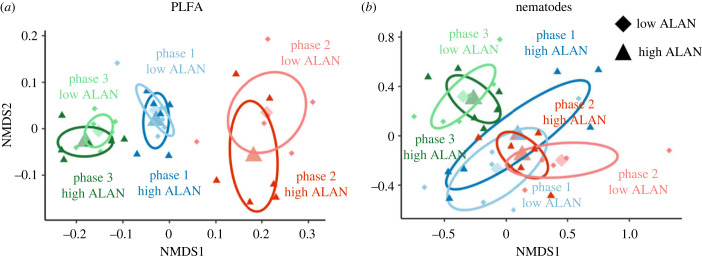
NMDS of (*a*) the microbial community based on fatty acid analysis (stress = 0.11) and (*b*) the nematode community (stress = 0.21) for three consecutive moon cycles (phases 1 to 3) and low (0.0014 Lux) and high (30 Lux) artificial light at night (ALAN).

The soil microbial community composition, as indicated by PLFA analysis, was not significantly affected by different levels of ALAN, but the phases differed significantly from each other (tables 2 and 3, figure 3*a*). With each subsequent phase, the amounts of AMF markers, fungal markers and one gram-negative marker increased (electronic supplementary material, figure S4). For the soil nematode community composition we observed a stronger effect, but it was only marginally significant, indicating a trend of ALAN×PHASE (table 2; figure 3*b*). Specifically, during the same phases, the nematode community under high and low ALAN never differed significantly from each other (table 3). By contrast, across all three phases, communities under low levels of ALAN significantly differed from each other, whereas the communities under high ALAN were similar to each other (homogeneous; table 3).

**Table 2.** NMDS results of a PERMANOVA testing the effects of artificial light at night (ALAN, low and high levels) and time as three consecutive lunar cycles (PHASE 1,2 and 3) and its interactions on the microbial community composition as indicated by phospholipid fatty acid (PLFA) analysis and the soil nematode community composition.

	d.f.	F	Pr(>F)
PLFA			
ALAN (A)	1,30	1.22	0.2926
phase (P)	2,30	33.38	0.0001***
A × P	2,30	0.54	0.7394
nematodes			
ALAN (A)	1,30	2.97	0.0074**
phase (P)	2,30	5.25	0.0001***
A × P	2,30	1.62	0.0714.

**Table 3.** Non-metric multidimensional scaling (NMDS) results of a PERMANOVA assessing the influence of artificiallight at night (ALAN) at low and high levels, time across three consecutive lunar cycles (PHASE, 1 to 3), and the interactions of ALAN x PHASE on microbial community compositon (phospholipid fatty acid – (PLFA) analysis) and soil nematode community composition.

		phase 2	phase 3	phase 1	phase 2	phase 3
		low ALAN	low ALAN	high ALAN	high ALAN	high ALAN
(*a*) PLFA						
phase 1	low ALAN	0.0134*	0.0027**	0.6169	0.003**	0.0018**
phase 2	low ALAN		0.0027**	0.0044**	0.2601	0.0018**
phase 3	low ALAN			0.0018**	0.0018**	0.7701
phase 1	high ALAN				0.0027**	0.0027**
phase 2	high ALAN					0.0027**
(*b*) Nematodes						
phase 1	low ALAN	0.0035**	0.0017**	0.1777	0.0502.	0.0031**
phase 2	low ALAN		0.002**	0.0148*	0.3958	0.002**
phase 3	low ALAN			0.0316*	0.0097**	0.5521
phase 1	high ALAN				0.3479	0.3543
phase 2	high ALAN					*0.0889.*

## 4. Discussion

This study provides evidence that artificial light at night (ALAN) has the potential to influence belowground communities and their functions. Over a period of 4.5 months, the effects of ALAN on soil microbial functioning and nematode community composition were observed to be small but detectable. These findings suggest that ALAN can rapidly extend its impact belowground. Consequently, the study highlights the broader range of potential consequences of light pollution on ecosystems than previously considered [2]. Furthermore, it serves as a call for future research to investigate the indirect effects of ALAN on areas that are not directly exposed to light, thereby expanding our understanding of the ecological implications of ALAN [50].

Notably, we discovered temporal variation in the consequences of ALAN for soil communities and functioning. The effects were most pronounced during phase 2 when plant biomass was overall highest and where ALAN significantly reduced plant biomass (932.1 ± 128.3 g dry plant biomass in phase 2 versus 331 ± 74.6 g dry plant biomass in phase 3 [34]). By contrast, in phase 3, the effect of ALAN was no longer significant, probably because the duration of plant growth was longer (nine weeks in phase 2 as compared to six weeks in phase 3; electronic supplementary material, figure S1), allowing plants to be exposed to the influence of ALAN for a longer period of time [34]. In phase 2 of the experiment, higher levels of ALAN were associated with significantly lower soil basal respiration, indicating decreased microbial activity. In parallel, the respiratory ratio decreased in phase 2 under high ALAN, indicating an increase in carbon use efficiency because the microbial biomass did not change while the basal respiration decreased. If microorganisms become more efficient then there can be a temporary imbalance in nutrient availability, potentially limiting plant growth and nutrient availability for other organisms. However, it is important to note that this pattern was only observed during the high plant biomass phase, and no direct links were identified. This suggests the involvement of other mechanisms that have yet to be discovered. Generally, the specific consequences of increased carbon use efficiency in soil can vary depending on environmental conditions, microbial community composition and the overall context of the ecosystem [51] and require more targeted analyses.

The strong effects of ALAN on plant biomass in phase 2 and the two soil microbial properties were also likely mediated by significant changes in soil water content. The higher plant biomass in phase 2 did not reduce soil water content, likely because plant communities with more biomass can reduce transpiration [52], create a more stable microclimate close to the soil surface [53,54] and be more efficient in water use than under high ALAN. This potential positive effect of plant communities on soil became smaller with time (phase 3), likely because the turnover of the plant community reduced the negative effects of ALAN on plant biomass [34]. Since soil water is a major driver of microbial activity [55] and soil water content was reduced under high ALAN, soil microbial activity also decreased. As we did not observe changes in the microbial community in phase 2 under high and low ALAN in the PLFA analysis, the suggested change in carbon use efficiency may rather reflect a difference in soil water content. The soil microbial biomass, in contrast, did not differ significantly at all. Measures of microbial biomass represent soil community response over larger periods of time, whereas soil microbial activity reflects conditions during soil sampling. Therefore, no change in the microbial biomass may reflect stable conditions. However, all other variables were strongly affected by PHASE, indicating that strong belowground community changes with time were common and changes in microbial biomass may be masked by other factors. That is, the positive influence of moist soil conditions that promoted increases in microbial biomass may have been counteracted by simultaneous increases in populations of microbial predators. For example, nematode communities, which were dominated by microbial-feeding nematodes, changed strongly over time, especially from phase 1 (lowest densities: 2.3 ± 1.8 individuals g^−1^ dry soil) to phase 2 (highest densities: 26.6 ± 15.5 individuals g^−1^ dry soil). The stable microbial biomass likely reflects the concomitant influence of soil moisture, plant biomass and strong grazing pressure by microbial-feeding nematodes, which can control soil microbial biomass and increase microbial activity [56].

Nematodes were more strongly affected by PHASE than by ALAN, suggesting that seasonal differences had a strong influence on our soil communities. This contrasts with the findings of Hölker *et al*. 2015 [8], who reported a loss of seasonal variation in freshwater sediment communities that were exposed to ALAN. Differences between these results could stem from the comparably smaller size of the study system (pots with 100 g soil in [8] compared to 246 kg soil in this study) and a longer duration of the study (1 year [8] compared to 4.5 months in this study), which could have increased the extinction probability of species in relatively simpler communities [57]. In our experiment, the population of plant-feeding nematodes showed a trend towards being higher under high ALAN, although the statistical significance was only marginal. Since stressed plants can be more susceptible to herbivory [58], the higher density of plant-feeding nematodes at high ALAN lends some support to the suggestion that ALAN stresses plant communities and makes them more vulnerable to belowground herbivory, although there is profound seasonal variability in the plant variables [34]. Although the proportion of plant-feeding nematodes was comparatively small in our study, their impact on productivity can be enormous [59], with the potential to enhance the negative impact of ALAN, especially when plants become more physiologically stressed. Additionally, under higher levels of ALAN, the channel index was higher, indicating that the fungal contribution to decomposition was increased. The channel index tends to increase with lower levels of freely available N and a higher amount of recalcitrant material [60]. Thus, changes in this index may indicate changes in the energy flux caused by ALAN. Long-term studies are needed to explore if the observed short-term trend of changing dominant decomposition and herbivory channels remain significant over time.

Although we hypothesized that ALAN effects would be mediated via plant biomass and plant diversity, plant diversity did not correlate significantly with any of the variables measured. This may be because the change in plant diversity (Shannon and Evenness) was significant in phase 2 but not accompanied by any significant loss in species richness [34]. The microbial and soil fauna responses that we recorded typically respond rapidly to abiotic and biotic changes. Yet changes in the plant community composition and plant diversity may take longer to materialize than those tested in this Ecotron study. By contrast to plant diversity, plant biomass was associated with changes in three measured variables (basal respiration, microbial biomass, and the channel index), but only in phase 3 and only when ALAN was high. Thus, we detected the direct effects of ALAN earlier (in phase 2) than the plant-mediated ones (phase 3), the phase without direct ALAN effects. The fact that we found these effects on microbial parameters and the channel index could indicate altered nutrient dynamics, e.g. due to changes in exudate quality and quantity. This may also suggest that effects of ALAN on belowground parameters are not mediated by plant biomass alone or not at the same time. For instance, the aboveground effects could be different from the belowground effects [61], and changes in the root-to-shoot ratio [62] or mycorrhizal associations and related functions can change in stressed plants [63], as well as the quantity and quality of rhizodeposition [64]. In addition, studies showed that belowground responses could be delayed for several weeks [65,66]. Therefore, follow-up studies should not only integrate soil organisms and functions to better explain the consequences of ALAN but also potential delays in belowground responses to better explain changes in above- and belowground community structure, ecosystem functioning, and stability.

We further hypothesized that different levels of ALAN lead to changes in the structure of soil microbial and nematode communities. We did not find any significant changes in the soil microbial community structure caused by different levels of ALAN, while the microbial community changed significantly over time in our experiment. This is in contrast to the community data of soil nematodes, where a significant effect of ALAN was found, as well as a marginal interaction of PHASE and ALAN. One explanation for this might be that effects were more pronounced in nematodes because of the higher taxonomic resolution of the data as compared to the microbial data (42 nematode taxa versus 14 fatty acid markers). PERMANOVA results of the single contrasts indicated that nematode communities under higher ALAN were generally more similar to each other than communities under lower ALAN, suggesting a higher degree of homogenization of community composition under high levels of ALAN. While the effects in this study were small and were studied for a short period of time, the effect of ALAN and the associated homogenization were shown to strengthen with time [8], with the potential to reduce stability and functioning in ecosystems [67]. Homogenization is a major threat to ecosystems, as uniform communities have a lower potential to withstand pressures such as droughts, pests and pollution because of reduced insurance and portfolio effects [67–69].

We expected to find a higher proportion of r-strategists and reduced food web complexity (indicated by the structure index) under high ALAN. R-strategists peaked in phase 2 but without significant differences between ALAN levels. Further indicators of stress (abundance of dauer larvae, cp-1 nematodes, maturity index, enrichment index, data not shown) were also not significantly affected by ALAN, suggesting that bacteria and bacterial-feeding nematodes did not benefit from any stress imposed by ALAN on plants or higher trophic levels [26]. By contrast to r-strategists, the Structure index and K-strategists tended to be higher at the end of the experiment. Nematode K-strategists have low fertility and a semi-permeable cuticula, making them particularly vulnerable to drought and chemical pollution [43]. Despite their relative sensitivity, their densities increased with time, probably reflecting succession but not increased levels of ecological stress. Nematodes at higher trophic levels have a wider range of food sources compared to those at lower trophic levels. They are often predators or omnivores, feeding on a variety of prey organisms including other nematodes or microarthropods. For instance, juvenile predatory nematodes start feeding on bacteria [43]. This trophic flexibility may allow them to adapt to changes in resource availability, making them less reliant on specific food sources that may be affected by potential changes caused by ALAN.

Given the ubiquity and increasing trend of ALAN across the globe, more and more studies are investigating its effects and are also extending into previously unexplored areas [2]. Nevertheless, the soil system has been neglected so far. This study shows that the soil system urgently needs to be considered in future ALAN research [4] due to its enormous biodiversity and functional importance. The next steps need to tease apart the influence of multiple drivers that simultaneously confront soil communities, such as changes in ALAN, soil moisture, plant productivity, diversity and phenology (above- and belowground), as all these factors can lead to substantial changes in the magnitude and timing of reponses from communities and ecosystems. In addition, we advocate taking a holistic view of potential changes imposed by ALAN on terrestrial ecosystems, ranging from changes in species to communities, as well as the magnitude and stability of functions [3] and fluxes across systems [70]. Our study showed that terrestrial ecosystem responses can, however, only be answered comprehensively when including the soil system.

## 5. Conclusion

This study emphasizes the potential of artificial light at night (ALAN) to impact biodiversity, species interactions and ecosystem functioning, not only directly but also indirectly by reaching the soil. Our findings reveal that the influence of ALAN can rapidly propagate into the soil within a few months of exposure. Further research is needed to investigate the long-term effects of ALAN on above- and belowground communities, including plants, microorganisms and higher trophic levels, as well as the potential for microevolution [71]. The observed changes in soil respiration and the respiratory ratio highlight that ALAN, as an indirect driver, can alter soil ecosystem functioning. Therefore, future predictions of soil functioning should consider a broader range of potential drivers, including ALAN, which may impact processes differently during day and night [17]. While ALAN offers benefits such as improved safety at night, it is also used for advertising and represents a symbol of the globalized world [72]. To mitigate system degradation and disruption of ecological cycles, it is important to reduce the extent of artificial light at night. Measures such as using light shields to direct light downwards and implementing time switches to limit ALAN duration [33] are of significance. This study provides novel insights into the widespread effects of ALAN on ecological communities and functions, even in systems that are not directly exposed to artificial light. Addressing the challenges associated with ALAN effects on soils and its potential impacts on issues like food and nutritional security requires interdisciplinary approaches. This involves raising awareness about the consequences of light pollution, implementing sustainable lighting practices, establishing outdoor lighting guidelines, promoting energy-efficient technologies and integrating ecological considerations into urban planning, agriculture and conservation strategies. Collaborative efforts among scientists, policymakers, industries and communities are essential for mitigating the negative effects of ALAN and ensuring a sustainable and resilient future for both societies and ecosystems.

## Data Availability

The datasets supporting this article have been uploaded as part of the electronic supplementary material [73].
